# Digital dentures in modern prosthodontics: Techniques, materials and clinical outcomes

**DOI:** 10.6026/973206300222342

**Published:** 2026-04-30

**Authors:** Neha Jogiswaran, Sumayya Surti, Navreet Kaur, Alekya Bommini, Navjot Singh, Aneeq Salik

**Affiliations:** 1 Department of Dentistry, Sri Ramakrishna Dental College and Hospital, Coimbatore, India; 2 Department of Dentistry, Isra Dental College, Hyderabad, Pakistan; 3 Department of Dentistry, Genesis Institute of Dental Sciences and Research, Ferozepur, Punjab, India; 4 Department of Dentistry, Sibar Institute of Dental Sciences, Guntur, India; 5 Department of Dentistry, CMH Lahore Medical College and Institute of Dentistry, Lahore, Pakistan

**Keywords:** Digital dentures, CAD/CAM, Additive manufacturing, subtractive milling, prosthesis accuracy

## Abstract

The integration of digital technologies into complete denture fabrication has raised important questions regarding their accuracy, clinical performance and reliability compared with conventional techniques. Digital denture workflows using CAD/CAM systems, intraoral scanning and design software have simplified fabrication procedures and improved predictability. Evidence indicates that digital and conventional impression techniques demonstrate comparable accuracy with digital methods performing better for short spans and conventional materials remaining more reliable for full-arch impressions. Both subtractive milling and additive 3D printing offer distinct advantages, with milled PMMA dentures showing superior strength and dimensional stability, while printed dentures provide greater design flexibility despite weaker bonding properties. Clinically, computer-engineered dentures reduce chairside time and post-insertion adjustments while achieving patient satisfaction and quality-of-life outcomes comparable to or better than conventional dentures, though further long-term comparative studies are required to establish standardized protocols.

## Background:

The digital evolution, which began with the introduction of computer-aided design/computer-aided manufacturing (CAD/CAM) in the 1970s for fixed restorations like crowns and bridges, promised a more streamlined and precise alternative [1]. In the early 1990s, CAD/CAM technology using 3D laser scanning and milling was first applied to complete dentures. The 2000s and 2010s saw major advancements with intraoral scanning, 3D printing and improved digital work flows [2]. Widespread adoption of digital dentures was initially slow due to high equipment costs and the need for clinicians and technicians to develop new digital skills [3]. Improvements in intraoral scanner accuracy and ease of use helped overcome many of these limitations [5]. These advancements ultimately enabled the adoption of both subtractive and additive manufacturing workflows in digital denture production [5]. Therefore, it is of interest to perform a review of various studies to show the accuracy and effectiveness of digital dentures.

## Historical evolution and adoption of digital denture technology and accuracy of digital impression techniques versus conventional methods:

The field of dentistry has undergone a major transformation with the integration of digital technologies, particularly in the domain of complete denture fabrication. For more than a century, complete dentures were produced through traditional analog workflows that required several clinical appointments and numerous manual laboratory steps. These steps-such as impression making, model casting, jaw-relation recording and acrylic processing-were prone to distortion and human error. The introduction of computer-aided design/computer-aided manufacturing (CAD/CAM) technology in the 1970s initially revolutionized fixed restorative dentistry by enabling a more efficient and precise workflow for crowns and bridges [1]. Its later extension to removable prosthodontics marked a significant departure from long-standing conventional methods. The early application of CAD/CAM to complete dentures began in the 1990s with systems relying on 3D laser scanning and milling. Substantial progress occurred in the 2000s and 2010s with the emergence of intraoral scanners, advanced design software and 3D printing technologies [2]. Despite these innovations, widespread adoption was slow. Barriers included high initial equipment costs and the need for clinicians and technicians to develop new digital competencies. The traditional hands-on approach gave way to a workflow requiring proficiency in digital scanning, virtual design platforms and operation of milling or printing equipment [3]. Over time, key technological advancements helped overcome these challenges. Improvements in the accuracy, reliability and user-friendliness of intraoral scanners enabled more predictable capture of soft and hard tissues in edentulous arches. As software interfaces became more intuitive, two primary manufacturing workflows solidified in clinical practice: subtractive and additive production [4]. Subtractive manufacturing mills dentures from pre-polymerized resin blocks whereas additive manufacturing (3D printing) constructs dentures layer-by-layer from photopolymer resins [5]. These technological improvements have driven a significant rise in digital denture adoption. Digital workflows now offer advantages such as reduced clinical appointments, enhanced reproducibility and the creation of permanent digital files that can be reused for repairs or remakes [6]. As advancements continue, digital dentures are becoming the standard of care in removable prosthodontics, enabling more predictable and streamlined treatment outcomes. This narrative review therefore examines the current status and ongoing developments in digital complete denture technology. A related area of investigation involves the accuracy of digital versus conventional impression methods. Evidence suggests that digital systems may achieve accuracy comparable to traditional impressions in fixed prosthodontics, making them a viable clinical alternative [7]. Some studies indicate superior accuracy with high-precision conventional materials, whereas others report improved marginal fit with digital impressions. Several authors, including Hasanzade, Tsirogiannis, Bandiaky, Papaspyridakos and Kong, concluded that both methods demonstrate similar accuracy overall. Factors affecting accuracy-such as undercuts and tooth angulation-may cause deviations in both anterior and posterior regions. Digital impressions have proven clinically acceptable for crowns, short fixed dental prostheses (FDPs) and implant-supported restorations, offering faster workflows. However, conventional techniques still appear more reliable for full-arch impressions due to cumulative inaccuracies in scanning larger spans [8, 9]. Studies comparing full-arch polyvinyl siloxane impressions with digital scans found improved mean accuracy for conventional methods, though differences between digital systems were not significant [10]. In contrast, some investigations reported significantly greater accuracy with intraoral scanners, demonstrated by reduced replica thickness in multiple measurement sites [11]. Therefore, both digital and conventional impression techniques have demonstrated strengths and limitations and ongoing research continues to refine their roles in contemporary prosthodontics.

## CAD/CAM design and manufacturing: Subtractive milling versus additive printing:

CAD/CAM technology in dentistry can be classified as either "Subtractive" or "Additive" manufacturing methods. Subtractive manufacturing methods include machining and milling (CAM) and laser ablation technologies, while additive manufacturing methods include 3D printing and laser melting [12]. In the field of digital dentures, both subtractive milling and additive printing (3D printing) are widely used, each with distinct advantages. Subtractive milling-typically using PMMA or pre-polymerized resin blocks-is valued for its precision, durability and smooth surface finish, making it suitable for long-term denture bases due to superior mechanical properties and fit accuracy [13]. Additive manufacturing allows for greater design flexibility, including internal structures and fine detail and is well suited for customization, rapid prototyping and short-term or interim dentures [14]. Although additive methods generally produce lower material waste, they often yield lower mechanical strength and increased surface roughness, requiring post-processing [15]. Current research suggests that the choice between these methods should depend on clinical need, cost, durability requirements and production scale and in many cases a hybrid workflow-using 3D printing for design and milling for final production-offers an optimal balance of efficiency and quality [16]. However, subtractive milling results in significant material waste and is limited in producing complex geometries, while studies show that milled dentures offer higher trueness and stability and 3D-printed dentures can achieve better internal adaptation in certain cases [17].

## Material properties, bonding strength and mechanical performance:

A digital denture refers to an innovation that encompasses devices, software programs, and associated materials used in the modern fabrication of dentures. One of the primary materials employed is industrially polymerized cross-linked polymethyl methacrylate (PMMA). This study analyzes the various cross-linked PMMA materials used in the fabrication of CAD/CAM complete dentures. CAD/CAM techniques are broadly categorized into subtractive (milling) and additive (3D printing) methods [18]. The integration of CAD-CAM technology and 3D printing in dentistry has enhanced both materials and workflows for denture fabrication. Benefits of CAD/CAM fabrication include reduced denture weight and lower resin volume, improving overall patient comfort. The choice of denture base resin-typically PMMA, light-curable methacrylate blends, or hybrid composites-impacts key physical properties such as flexural strength, modulus, fatigue resistance, water sorption, and hardness. PMMA remains the most commonly used material due to its favorable characteristics including esthetics, low cost, low water absorption, biocompatibility, ease of processing, and repairability. However, it also presents limitations such as porosity, residual monomer release, brittleness, and inconsistent thickness. Milled CAD/CAM PMMA bases generally exhibit higher flexural strength (120-146 MPa) compared to 3D-printed and conventionally heat-polymerized resins [19]. Recent advances in additive manufacturing have improved the fabrication of 3D-printed complete dentures; however, research consistently shows that 3D-printed denture bases demonstrate lower bond strength and inferior mechanical properties compared with both conventional heat- polymerized PMMA and CAD/CAM-milled PMMA bases. Current digital workflows allow denture teeth to be produced separately-either through milling high-strength PMMA blocks or printing microfilled resin teeth-and later bonded to the printed base. This modular fabrication enhances esthetics, customization, and precision. Bonding is typically achieved using light-polymerized resin adhesives or mechanical surface treatments, including air abrasion, chemical conditioning, and micromechanical retention, all of which improve interfacial adhesion and reduce the likelihood of tooth debonding or base fracture [20]. Recent research comparing denture teeth bonded to heat-polymerized, CAD/CAM-milled, and 3D-printed denture base resins shows consistent differences in mechanical performance. Studies report that heat-cured PMMA bases exhibit the highest fracture toughness and bond strength, while CAD/CAM - milled and especially 3D-printed resins show reduced interfacial durability. Multiple investigations evaluating four-point bending, notched-beam KIC testing, and flexural bond strength found that 3D-printed DBRs have the lowest fracture toughness, with values often less than half those of heat-cured PMMA. CAD/CAM milled resins show intermediate results but remain significantly weaker than conventionally processed bases. Artificial aging through thermal cycling consistently decreases bond strength across all materials, with printed resins showing the largest reduction. Failure mode analysis commonly reveals cohesive failures in milled resin groups, whereas adhesive failures dominate in 3D printed and heat-cured groups, indicating weaker interfacial adhesion. Overall, studies conclude that heat-polymerized PMMA remains the most reliable denture base for bonding to commercial acrylic or composite teeth, while printed materials show the poorest and least durable adhesion [21]. Recent studies confirm that polymerization shrinkage and dimensional inaccuracy remain key limitations of conventionally processed denture bases. In contrast, CAD/CAM-milled PMMA uses pre-polymerized, industrially polymerized PMMA discs, which eliminates in-process shrinkage and results in superior dimensional stability, improved fit accuracy, and enhanced mechanical performance. The highly dense and homogeneous structure of pre-polymerized PMMA contributes to better adaptation to the underlying tissues and reduces internal stresses typically associated with chemically activated polymerization [22].

## Clinical effectiveness, patient satisfaction and quality of life outcomes:

Computer-engineered complete dentures (CECDs) have shown strong clinical effectiveness, high patient satisfaction and positive quality- of-life outcomes. In comparison to conventional dentures, CECDs need less post-insertion adjustments and follow-up visits. Saponaro *et al.* [23] found that 6 of 48 patients required no adjustments, 16 needed one visit, fewer than 16 required two visits and 25% (n = 12) needed three or more. Similarly, Bidra *et al.* [24] reported that only 3.3% of dentures required modification after a year. These findings indicate that CECDs not only streamline the treatment process but also enhance patient comfort and reduce the clinical workload. In addition to reduced adjustment needs, CECDs offer shorter treatment times and greater overall efficiency compared to conventional dentures, which often require follow-up within 24 hours and up to three adjustment visits [25, 26]. Their digital fabrication process provides several practical advantages, including easier manufacturing, reduced chair time and the ability to store data digitally for quick replacement if a denture is lost or damaged [27]. Typically, CECDs can be completed in just two appointments, saving approximately one hour of clinical time and five hours of laboratory work. Importantly, Peroz *et al.* [28] observed that these efficiencies translate into measurable improvements in patients' oral health-related quality of life. Furthermore, research has demonstrated that milled, pre-polymerized polymethyl methacrylate (PMMA) bases used in CECDs can offer equal or even superior retention compared to conventional heat-polymerized bases. Digital dentures also tend to have a more accurate base fit, better clinical retention and fewer denture-related traumatic lesions, while CAD-CAM-fabricated monolithic prostheses deliver exceptional precision and reproducibility [29]. Overall, patients receiving CECDs have reported high satisfaction levels, with retention and comfort comparable to, or exceeding, those of conventional dentures [30]. Supporting this, Inokoshi *et al.* [31] found that satisfaction scores were similar between digital and traditional dentures. Additionally, the ability to digitally store patient data allows den^32^].

## Limitations, cost effectiveness and future research directions:

Despite these advantages, CECDs are not without limitations. One major drawback is the absence of a clinical try-in stage, which prevents the evaluation of aesthetics and phonetics prior to final fabrication and may influence patient satisfaction. The fabrication process also demands specialized training and experience, requiring clinicians to invest time in assessing digital previews and maintaining consistent communication with the dental laboratory to ensure successful outcomes [33]. Nevertheless, digital dentures demonstrate clear advantages in terms of efficiency and cost. A comparative study reported that the average chairside time for digital dentures was 154.31 ± 13.19 minutes, significantly less than the 218.00 ± 20.75 minutes required for conventional dentures (p < 0.0001). Laboratory costs were also lower for digital dentures (€378.79 ± 137.46 vs. €459.15 ± 63.72, p = 0.0059) [34]. These findings suggest that CECDs not only improve workflow efficiency but also offer potential cost savings for both clinicians and patients. Although the outcomes associated with digital dentures are promising, current research is still limited by small sample sizes, short follow-up periods and variations in clinical protocols. To establish stronger evidence, future studies should focus on larger randomized controlled trials assessing long-term parameters such as oral health- related quality of life, prosthesis survival and biological complications. Comparative studies between milled and three-dimensional printed dentures are also essential to clarify differences in performance, cost-effectiveness and patient adaptation. In addition, advancements in denture materials, the integration of artificial intelligence into prosthetic design and the development of reliable digital archives for rapid denture reproduction present exciting directions for the future. As digital technology continues to evolve, consensus statements highlight the need for standardized clinical protocols and multicenter studies with long-term follow-up to validate and refine these innovative digital approaches [35, 36 and 37]. Digital complete denture workflows simplify clinical and laboratory procedures by reducing the number of appointments and enhancing standardization. Their findings emphasize that CAD/CAM dentures offer improved predictability and reproducibility compared to conventional fabrication techniques [38]. CAD/CAM complete dentures demonstrate high accuracy and favorable clinical outcomes, particularly in terms of fit and dimensional stability. The study supports the reliability of digital denture fabrication as a viable alternative to conventional methods [39]. Compared digital and conventional complete dentures and found comparable clinical performance and patient satisfaction between the two approaches. The authors concluded that digital dentures provide clinical outcomes similar to conventional dentures while offering advantages in efficiency and workflow optimization [40].

## Conclusion:

Digital denture technology has advanced substantially through improvements in CAD/CAM workflows, material properties and intraoral scanning, offering more predictable, efficient and patient-centered prosthetic outcomes. Evidence demonstrates that computer-engineered complete dentures provide strong clinical performance, high patient satisfaction and meaningful reductions in treatment time and cost compared to conventional methods. Continued research with larger, long-term clinical trials is essential to further validate these benefits, optimize digital materials and workflows and support the widespread adoption of fully digital denture protocols.

## Advancement to knowledge:

This article provides an updated synthesis of recent evidence on digital complete dentures, comparing CAD/CAM workflows with conventional techniques. It highlights clinical accuracy, efficiency and manufacturing advances (milling vs. 3D printing), supporting evidence-based adoption and future standardization in prosthodontic practice.
